# Eosinophilic Enteritis with Ascites in a Patient with Overlap Syndrome

**DOI:** 10.1155/2009/734206

**Published:** 2009-05-24

**Authors:** Spyros Aslanidis, Athina Pyrpasopoulou, Kostas Soufleris, Eirini Kazantzidou, Stella Douma

**Affiliations:** ^1^2nd Propedeutic Department of Internal Medicine, Hippokration General Hospital, 54643 Thessaloniki, Greece; ^2^Radiology Department, Hippokration General Hospital, 54643 Thessaloniki, Greece

## Abstract

Gastrointestinal involvement is frequent in patients with systemic lupus erythematosus (SLE). Eosinophilic gastroenteritis, however, has only rarely been described in rheumatological conditions, despite its reported connection to autoimmune diseases, such as hypereosinophilic syndrome, vasculitides, and systemic mastoidosis. It presents typically with abdominal pain and diarrhea and is only exceptionally associated with ascites. Diagnosis can be problematic, as several other clinical conditions (malignancies, infection/tuberculosis, and inflammatory bowel diseases) have to be ruled out. It is basically a nonsurgical disease, with excellent recovery on conservative treatment. We report the rare case of a young woman with overlap syndrome who presented with abdominal pain and ascites. The diagnosis of eosinophilic enteritis was made based on clinical, radiological, and laboratory criteria. The patient was treated with corticosteroids with excellent response.

## 1. Introduction

Gastrointestinal involvement is frequent in patients with systemic lupus erythematosus (SLE). In a series of 201 patients with childhood-onset SLE the most frequent symptom was abdominal pain. It was mostly related to lupus involvement, especially ascites, and pancreatitis, more rarely to treatment-induced events or infection and never to events unrelated to SLE [1]. Eosinophilic gastroenteritis has only rarely been described in rheumatological conditions, such as rheumatoid arthritis and systemic lupus erythematosus [2–6], despite its reported connection to autoimmune diseases, such as hypereosinophilic syndrome, vasculitides, and systemic mastoidosis. It presents typically with abdominal pain and diarrhea and is only exceptionally associated with ascites. Diagnosis can be difficult, as several other clinical conditions (malignancies, infection/tuberculosis, and inflammatory bowel diseases) have to be ruled out. Differential diagnosis from lupus enteritis (gastrointestinal vasculitis), a common cause of acute abdominal pain associated with small bowel wall thickening and ascites, involves exclusion of mesenteric ischemia [7].

We report the rare case of a young woman with overlap syndrome who presented with abdominal pain and ascites. Based on clinical, radiological, and laboratory criteria, the diagnosis of eosinophilic enteritis was made. The patient was treated conservatively with corticosteroids with excellent response.

## 2. Case Presentation

A 24-year-old woman presented with abdominal pain and vomiting. She was previously well till 24 hours earlier when she developed diffuse abdominal pain, nausea, and vomiting, with gradual worsening. On clinical examination, the patient was afebrile, tachycardic (108/min), with diffuse tenderness, guarding of the entire abdomen and abdominal distension; there were no active bowel sounds. 

The patient's medical history included heterozygous *β*-thalassemia and a 6-year history of connective tissue disease with overlapping features of systemic lupus erythematosus and limited scleroderma, with positive antinuclear-ANA, antiribonucleoprotein-anti-RNP, anti-DNA and anti-*β*2-glycoprotein antibodies for which she was currently on methotrexate (10 mg/week), hydroxychloroquine (400 mg/day) and low dose aspirin. Two months prior to this episode she reported similar symptomatology, which responded well to “classical” treatment (ranitidine and metoclopramide).

**Figure 1:**
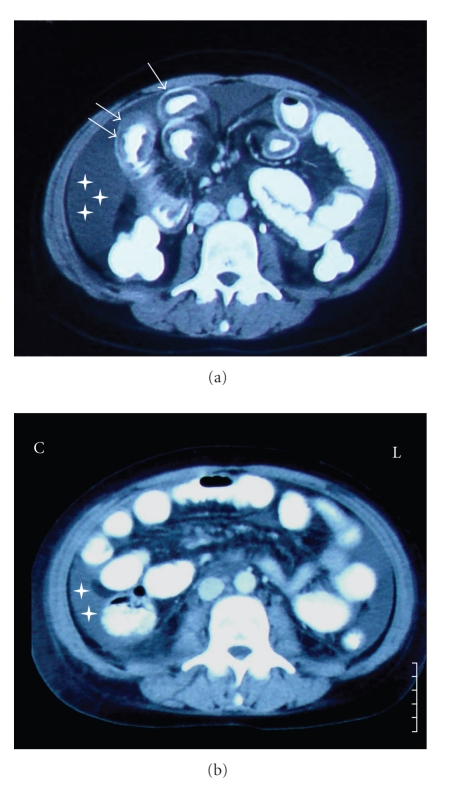
Computed tomography scan of the abdomen performed. (a) On day 3 of the patient's admission, 4 days after initiation of symptoms. Arrows: thickening/dilatation of the intestinal wall, stars: ascitic fluid. (b) Four days after initiation of treatment, with marked improvement of the intestinal morphology, and moderate reduction of the amount of ascitic fluid.

On the 3rd day after admission her findings persisted; computed tomography (CT) scanning of the abdomen revealed dilatation and marked contrast-enhanced thickening of the small intestinal wall, mainly ileum; other parts of the small intestine (jejunum) and the large intestine were unaffected. A substantial amount of ascitic fluid was also present (Figure 1(a)). Large vessels were patent, with no signs of occlusion, venous thrombosis, intestinal ischemia, or intestinal obstruction; no pathological lymph nodes were observed.

The patient was referred to our hospital for further evaluation and treatment.

Diagnostic paracentesis of the ascitic fluid showed low SAAG (serum/ascites albumin gradient), elevated lactic dehydrogenase (LDH), and presence of eosinophils (23% of 800 WBC/*μ*L, 52% neutrophils, and 15% lymphocytes) in the ascitic fluid without peripheral eosinophilia; the number of peripheral eosinophils rose to 13% (1100/*μ*L) on the 4th day of her hospitalization. The PPD (tuberculin, purified protein derivative) skin test and the cultures of the ascitic fluid were negative. 

The diagnosis of eosinophilic enteritis was made based on clinical, laboratory, and radiological criteria. Endoscopy was not undertaken because of (a) the patient's severe clinical condition (peritonism and marked intestinal dilatation), (b) the difficulty in accessing the affected area (ileum), and (c) the strong propability of negative biopsies in this particular (subserosal) type of involvement [8].

The patient was treated with IV prednisolone (1 mg/kg daily for 7 days and gradual tapering) and IV cefotaxime, with rapid resolution of her symptoms and considerable clinical improvement. A subsequent scan, performed 4 days after initiation of treatment showed significant normalization of the morphology of the intestinal wall with moderate reduction of the ascitic fluid (Figure 1(b)). Five months later, the patient was in excellent clinical condition under a maintenance dose of methylprednisolone (2 mg every other day), soon to be discontinued.

## 3. Discussion

Eosinophilic gastroenteritis is a heterogeneous and uncommon disease, affecting children as well as adults, whose pathogenesis and etiology are not well understood. The diagnosis of eosinophilic gastroenteritis is based on a high degree of suspicion and represents a challenge to the physician, not only because of its rarity, but also because of the variety of clinical manifestations. No definite criteria exist; diagnosis is based on a combination of clinical, radiological, and histological findings. Clinically, the presence of gastrointestinal symptoms, the lack of evidence of parasitic or extraintestinal eosinophilic disease and the exclusion of intestinal lymphoma, Crohn's disease or other tumors, nodular and irregular thickening of the folds usually in the distal stomach and proximal small bowel on CT [9, 10], and the histologically proven intense eosinophilic infiltration in any of the different layers of at least one site of the gastrointestinal tract suffice for the diagnosis [11–13]. Eosinophilic gastroenteritis is basically a nonsurgical disease, with excellent recovery on conservative treatment [14, 15]. Steroids are usually indicated as first line therapy for patients with nonallergic eosinophilic gastroenteritis and provide prompt and effective relief of symptoms within a few days to weeks. Most experts recommend doses similar to those used in inflammatory bowel disease (1-2 mg/kg per day), tapered over a period of 3-4 months to induce remission. Relapses however are frequently noted during steroid tapering or after discontinuation, necessitating repeated steroid therapy [15]. Therefore, long-term follow-up is warranted, despite initial rapid improvement, since there is no agreement on the duration of treatment.
